# Particle Image Velocimetry Method for Prediction Hydrodynamic Conditions during Leaching Process on the Basis of Sn–NaOH System

**DOI:** 10.3390/ma14030633

**Published:** 2021-01-29

**Authors:** Adam Cwudziński, Bernadeta Gajda

**Affiliations:** Department of Metallurgy and Metals Technology, Faculty of Production Engineering and Materials Technology, Czestochowa University of Technology, Armii Krajowej 19, 42-201 Czestochowa, Poland; kmitm@wip.pcz.pl

**Keywords:** particle image velocimetry (PIV), leaching process, hydrodynamics, Sn–NaOH system

## Abstract

In leaching processes controlled by diffusion and convectional transport of mass, the hydrodynamic structure formed in the reactor’s working volume is an additional factor affecting the process. This research work presents results related to hydrodynamic structures developing in batch reactors, different in shape, recorded by means of the particle image velocimetry (PIV) method. The movement of the distilled water and leaching solution was analyzed during investigations. Next, the system hydrodynamics and the process of tin leaching were analyzed. Finally, the leaching is affected by the reactor geometry and the hydrodynamic structure developed in its working volume, especially when a convectional or diffusion mass transport decides the process efficiency.

## 1. Introduction

Continuous technological progress requires deliveries of metals, of which the natural resources in the Earth’s crust are being successively depleted. Hence, a very important element of metallurgy consists in the effective recovery of metals from products withdrawn from use or from the process waste [1,2,3,4,5,6,7,8,9]. Metallic scrap most frequently comprises a few precious metals; therefore, methods for their recovery are of a many-stage and selective nature. Methods of preliminary separation are used in hydrometallurgical metal recovery processes, as well as enrichment, leaching, resolution, and then separation [10,11,12,13,14]. Leaching is a stage effectively limiting the process of metals recovering from secondary materials, which is carried out in batch or flow reactors. The efficiency of leaching depends, inter alia, on the method and intensity of mixing, primarily on the composition and degree of leached material disintegration, the type and concentration of the leaching agent, and also on the time, temperature, or pressure in the system [15,16,17,18,19,20]. In leaching processes controlled by diffusion and convectional transport of mass, the hydrodynamic structure formed in the reactor’s working volume is an additional factor affecting the process. The leaching agent flow in the immediate vicinity of the leached agent affects the boundary layer thickness, where the mass transfer is controlled by the diffusion. Therefore, intensive mixing of the continuous medium enables increasing the productivity of metals transfer from the solid to the liquid phase. The hydrodynamic structure in the reactor’s working volume is a resultant of the reactor’s geometry, location of the propeller blades, its design and rotational speed, location of the heating system, physical and chemical properties of the system substrates, and the physical shape of the leached agent. The Sn–NaOH system is one of the hydrometallurgical systems in which mass transport plays an important role in process efficiency [20,21]. Tin is a metal using to produce special alloys and for soldering or coating. Therefore, tin, like major industrial metals with abundance in the earth’s crust below 0.0002%, required additional insight into fundamental knowledge on tin metallurgy [22,23]. This research work presents results related to hydrodynamic structures developing in batch reactors, different in shape, recorded by means of the particle image velocimetry (PIV) method [24,25,26,27]. Particle image velocimetry is the non-invasive method for predicting hydrodynamics wherever the testing objects and fluids are transparent. The impact of system geometry on the process of metallic tin dissolution in a 0.5M NaOH solution was verified.

## 2. Testing Objects and Methodology

**Figure 1:**
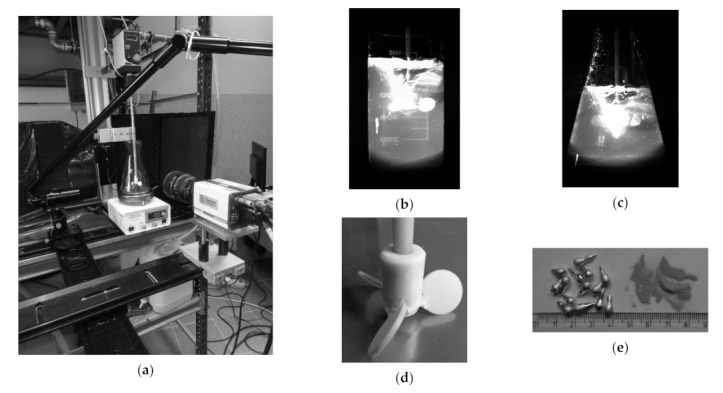
Components of study: (**a**) research stand with reactor no. 2, PIV system and stirrer-heater system; (**b**) reactor no. 1; (**c**) reactor no. 2; (**d**) propeller sketch; (**e**) Sn materials in the form of drops and powder.

The agitation leaching process is one of the stages which is used in the industrial hydrometallurgy of Ni, Co, Cu, Fe-Ti, Zn; therefore, still additional investigations for better process cognition are required [28,29]. Figure 1a presents a test stand for analysis of fluid–solid system hydrodynamics, which consisted of a heating station with a thermostat, an overhead propeller with rotations control (R50D type, CAT M. Zipperer GmbH, Ballrechten-Dottingen, Germany), a camera, an optical system leading a laser beam, a laser, and a computer with a controlling-recording system. The propeller was equipped with three circle propeller blades. The diameter of each blade was 0.018 m. The diameter of the propeller shaft was 0.006 m. Additionally blades were angled at 50 degrees (Figure 1d). Because of different type of leaching reactors, two characteristic vessels were chosen for investigations [30,31,32,33,34,35]. Glass cylinders (base 0.09 m in diameter, 0.18 m high) and glass cones (base 0.125 m in diameter, neck 0.07 m in diameter, 0.2 m high) of 1 L nominal volume were the chemical reactors (Figure 1b,c). The amount of leaching agent was 800 mL, and the amount of tin was added to the system at a ratio of 1:100. Tin with 99.9% purity was added in the form of drops (approximately 1 cm long, 0.5 cm in diameter) and powder (modal value: 1.6 μm in diameter). Figure 1e presents tin in the form of drops and powder. The tests were carried out for two levels of the propeller blade immersion in the leaching liquid, i.e., 500 mL (top position) and 200 mL (bottom position).

For prediction hydrodynamics inside both reactors, a double frame sCMOS camera (DantecDynamics, Skovlunde, Denmark) was used. A camera and a double-cavity laser of pulse energy of 200 mJ and a wavelength of 532 nm with an optical light knife system with a light beam were used during laboratory trials. For the analysis of the vector flow field, the DynamicStudio software (Version 6.9.6) with the particle image velocimetry (PIV) module was employed. Seeding in the form of 0.05 g glass borosilicate balls of a density of 1100 kg/m^3^ (± 50 kg/m^3^) and an average diameter from 9 to 13 μm was introduced to the water or 0.5M NaOH solution (Lavision GmbH, Gottingen, Germany). During a single measurement, 150 double frames with 15 Hz frequency were recorded, which provided a basis for obtaining the vector flow field. The time between frames was 5153, 553 and 53 μs, respectively, for 49, 300 and 500 rpm. The measurement during each case was repeated 5 times (separate trials) at intervals of 4 min during distilled water experiments and 4 times at specific times 5, 15, 20 and 30 min for the NaOH-Sn system. In the next stage of analysis, the recorded glass ball motion was transformed into the vector flow field.

Results of the horizontal and vertical velocity obtained during the PIV analysis were recorded from two measuring lines, one horizontal situated 1 cm above the reactor’s bottom and one vertical situated parallel to the propeller shaft at a distance of 3.4 cm. In the first stage of tests, the distilled water was the model liquid to evaluate hydrodynamic conditions originating in both analyzed reactors. Three rotational speeds, i.e., 50, 300, and 500 rpm were tested during the distilled water study (Table 1). The movement of the leaching solution (NaOH) was analyzed in the second stage of tests (Table 2, cases: 13–16). Next, in the third stage, the system hydrodynamics and the process of tin leaching were analyzed at the same time (Table 2, cases: 17–22). The process of metallic tin dissolution in the form of drops and powder was carried out at two temperatures, i.e., 293 and 323 K, and a pressure of 1 atm. The higher temperature guaranteed that the process will be run in the area controlled by the mass transport only [20,21]. Samples of the leaching solution were manually taken during experiments, at specified intervals after 5, 15, 20, 25, and 30 min of the process duration. The taken samples were diluted and the tin content in them was determined by means of an Agilent MP-ES 4200 emission spectrophotometer (Agilent, Santa Clara, CA, USA), in which the microwave-induced nitrogen plasma, at a temperature of 5000 K, was used to excite elements. The spectrophotometer was equipped with an Agilent 4107 nitrogen generator, collecting nitrogen directly from the air, supplied by means of a compressor. All cases of testing variants for distilled water and NaOH solutions, considered in this research work are shown in Table 1 and Table 2, respectively. The cycle of experiments presented in Table 1 was the same for both reactors considered in this research work.

## 3. Results and Discussion

### 3.1. Hydrodynamics in the Water

**Figure 2:**
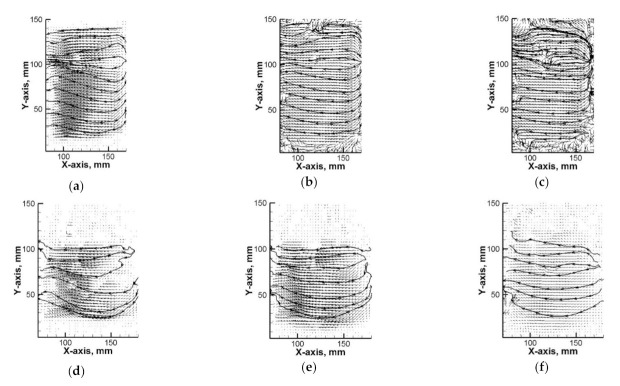
Velocity flow field of water (particle image velocimetry (PIV) experiment) for top position of propeller blades: (**a**) reactor no. 1 (50 rpm); (**b**) reactor no. 1 (300 rpm); (**c**) reactor no. 1 (500 rpm); (**d**) reactor no. 2 (50 rpm); (**e**) reactor no. 2 (300 rpm); (**f**) reactor no. 2 (500 rpm).

Figure 2 presents vector maps of water movement in both reactors on a plane perpendicular to the bottom, shifted towards the camera by 1 cm from the propeller’s axis. In both reactors, a zone of intensive mixing existed in the place of the propeller blade’s location. In the case of reactor no. 1, falling streams develop above the propeller blades while rising streams under the propeller blades (Figure 2a). Instead, the streams inflowing to the plane of measurement have an opposite sense, rise above the propeller blades and fall to the bottom in the zone under the propeller blades. A similar hydrodynamic system forms in reactor no. 2, where the distribution of streams under the propeller blades is more parabolic in nature (Figure 2d). For the considered reactors, it is also characteristic that with a growing rotational speed of the propeller, the horizontal arrangement of flow streams becomes normalized (Figure 2b,c,e,f).

**Figure 3:**
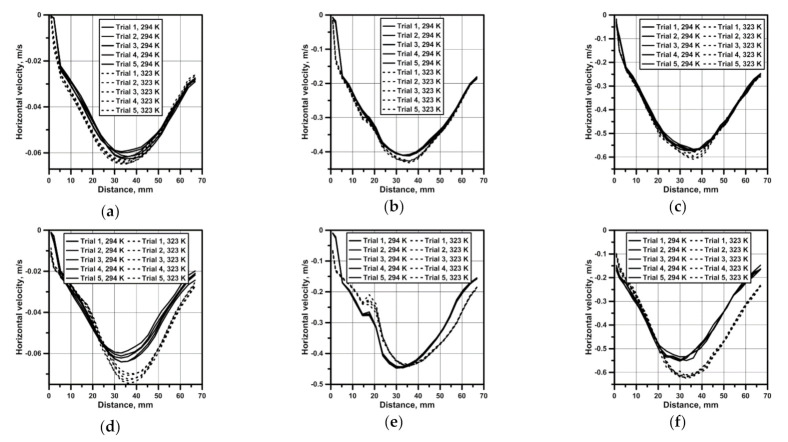
Velocity profile in reactor no. 1 and from horizontal line: (**a**) bottom propeller position (50 rpm); (**b**) bottom propeller position (300 rpm); (**c**) bottom propeller position (500 rpm); (**d**) top propeller position (50 rpm); (**e**) top propeller position (300 rpm); (**f**) top propeller position (500 rpm).

**Figure 4:**
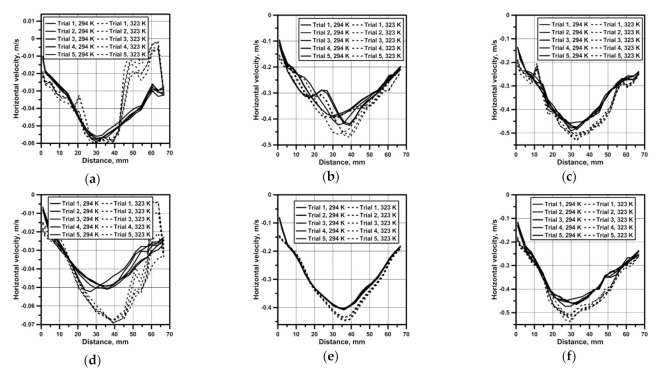
Velocity profile in reactor no. 2 and from horizontal line: (**a**) bottom propeller position (50 rpm); (**b**) bottom propeller position (300 rpm); (**c**) bottom propeller position (500 rpm); (**d**) top propeller position (50 rpm); (**e**) top propeller position (300 rpm); (**f**) top propeller position (500 rpm).

For all the considered testing variants, the measurements of the flow field were performed in the measuring plane, which was next to the basis for the quantitative analysis of velocity fields of the continuous medium flow in the reactor. Figure 3 presents results of the horizontal water velocity component for reactor no. 1, which confirm a parabolic velocity distribution also in the zone situated 1 cm above the bottom. On the propeller’s axis, the maximum speed was approx. 0.062 m/s for the top and bottom propeller blades location (Figure 3a). In the top propeller blades location variant, with the system heated from the bottom, there was a clearer modification of the hydrodynamic system resulting from movements related to the heat convection for all considered rotational speeds of the propeller (Figure 3d–f). A six- and ten-time increase in the propeller’s rotational speed caused a linear increase in the maximum velocity of the continuous medium in the reactor (Figure 3b,c,e,f). In the case of reactor no. 2, featuring a larger base and hence a larger area related to the heat flow, the hydrodynamics modification in variants with 50 rpm mixing is clear for both propeller’s locations (Figure 4a,d). For reactor no. 2, despite the fact that the propeller blades both in the bottom and the top position were situated closer to the bottom, the obtained maximum velocity values were smaller than in the case of reactor 1. Like in the case of reactor no. 1, parabolic velocity distribution in the bottom zone was obtained as well (Figure 4a–f).

**Figure 5:**
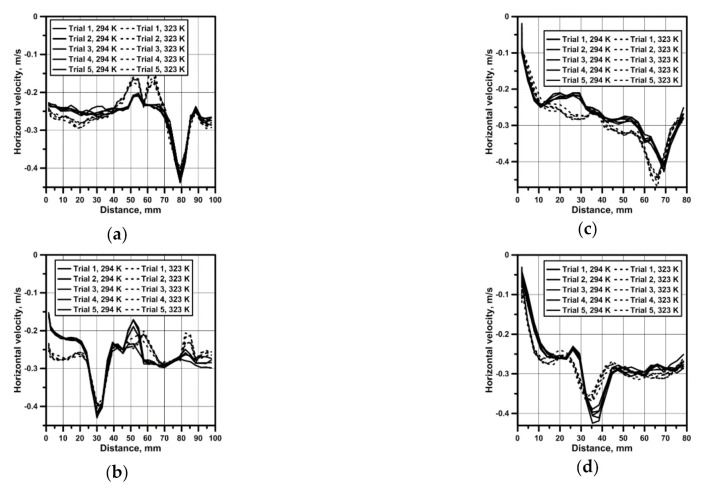
Velocity profile from the vertical line: (**a**) bottom propeller position (500 rpm) and reactor no. 1; (**b**) top propeller position (500 rpm) and reactor no. 1; (**c**) bottom propeller position (500 rpm) and reactor no. 2; (**d**) top propeller position (500 rpm) and reactor no. 2.

Figure 5 presents values of the vertical velocity on a vertical measuring line to assess the fluid behavior on individual depth levels for a rotational speed of the propeller equal to 500 rpm. The origin of the system of coordinates in Figure 5 means a free surface of the water. For both reactors, characteristic velocity peaks, featuring an average value of approx. 0.4 m/s, exist in places of the propeller blades location. A common feature of both reactors consists in the fact that at a bottom propeller blade’s location, slightly higher maximum velocities are obtained in the bottom zone, where particles of leached materials, featuring a density higher than the leaching agent density, situate themselves. Moreover, sometimes particles sizes making their movement in the working space of the reactor impossible; therefore, not all of the particles exist in the zone of maximal fluid velocity. The presented results of experiments for both reactors also confirm good repeatability of consecutive measurements, providing a reliable image of the hydrodynamic system shaped in both reactors. Results related to the hydrodynamics developing in both reactors show a limited area of propeller’s impact in the analyzed reactors’ working spaces.

### 3.2. Hydrodynamics in the NaOH Solution

**Figure 6:**
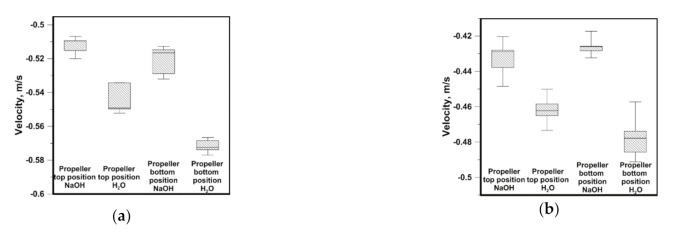
Maximal velocity from horizontal line: (**a**) reactor no. 1; (**b**) reactor no. 2.

**Figure 7:**
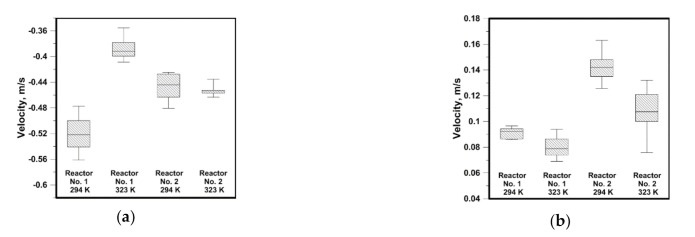
Maximal velocity from the horizontal line—Sn in the form of drops: (**a**) horizontal component of velocity; (**b**) vertical component of velocity.

**Figure 8:**
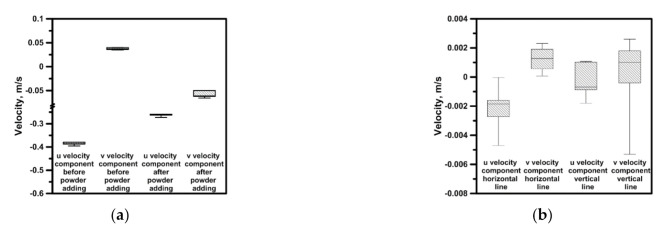
Maximal velocity—Sn in the form of powder: (**a**) reactor no. 1 with stirring (500 rpm); (**b**) reactor no. 1 without stirring.

**Figure 9:**
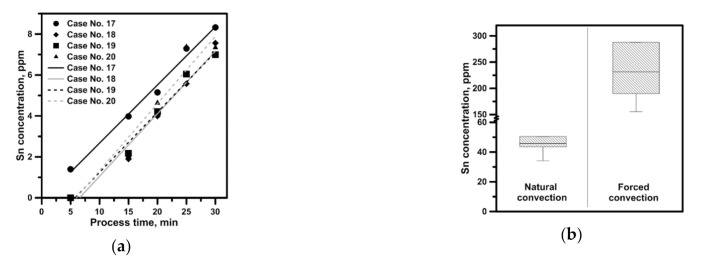
Sn concentration in the NaOH: (**a**) Sn drops; (**b**) Sn powder after 30 min of process.

The next stage of tests comprised verification of the fact, to what extent the results obtained for the distilled water can differ from the leaching agent, i.e., NaOH solution. The results from Figure 6, Figure 7, Figure 8 and Figure 9 are presented in the form of box-wisher plots where centerline means median, box edges mean quartiles, and whiskers means minimum/maximum values. Figure 6 presents results of the maximum flow velocities for the distilled water and NaOH solution obtained on the horizontal measuring line in both reactors and for both propeller blade locations. In the case of reactor no. 1, the difference between the water and NaOH solution velocities was 0.02 m/s and 0.01 m/s, for the top and bottom propeller’s location, respectively. Instead, a higher difference was recorded for reactor no. 2 in the case of the bottom propeller’s location, equal to 0.05 m/s. The results obtained for the NaOH solution featured smaller velocity values obtained in reactors’ working spaces. However, the presented results confirmed higher velocities obtained in reactor no. 1 and at the bottom propeller’s location.

### 3.3. Hydrodynamics and Leaching Process in the Sn–NaOH System

The introduction of an additional factor, e.g., in the form of solid particles, to the hydrodynamic system modifies the flow structure; therefore, the process of tin dissolution in both considered reactors was carried out for a rotational speed of the propeller of 500 rpm. The hydrodynamics of a heterogeneous system is particularly important for reactions, which are controlled by the mass transport to the reaction limit. Hence, the more intensive mixing in the system, i.e., higher velocities of the continuous medium, the more efficient is the mass transport. Limited areas of the increased leaching agent flow velocities exist in the considered systems. Therefore, the mass transport for the analyzed reactors will depend on the position of the leached agent in the reactor’s working volume. In the next stage of testing, 8 g portions of tin drops were introduced to the reactor after stabilization of hydrodynamic conditions and temperature (variant for 323 K). During most experiments the drops were not susceptible to the vortical movement caused by the propeller and situated themselves on the bottom, at the edge joining the reactor’s bottom and side wall. In the outermost reactor’s bottom zones the velocities ranged from 0.03 to 0.1 m/s, at a rotational speed of 500 rpm. Figure 7 presents values of the maximum velocity reached on the level of horizontal measuring line during the process of Sn dissolution in NaOH. In reactor no. 1 the smallest velocities were recorded at a temperature of 323 K. While in the same reactor the velocity was highest at a temperature of 294 K. In the conical reactor, in processes carried out at both temperatures, the velocity values were similar. The highest maximum values for the vertical component of approx. 0.142 m/s were obtained in the cone reactor at a definitely greater spread of the velocity values range. Instead, in the cylinder reactor the average velocities were smaller. In the next stage of testing the Sn granules were replaced with powder to verify, to what extent the process will be more efficient if substrates react in the zone of increased velocities. Figure 8a presents results of the velocity obtained on the horizontal measuring line level before and after the powder introduction. For the horizontal component, after the powder introduction to the system, the velocity reduction was recorded due to the continuous medium thickening with the solid metallic phase. While for the vertical component the vector direction changed. After the powder introduction the vertical component of velocity was declining in nature. In addition, to obtain the image related to the mass transport only in the area of natural convection, experiments were carried out without the propeller’s use. Reactor no. 1 was heated up to a temperature of 323 K of the leaching liquid. The tin powder was introduced to the reactor once the leaching liquid was heated and maintained for a specified time at a temperature of 323 K. The convectional movement of the liquid caused by the temperature gradient developed the hydrodynamic structures permanently modifying themselves during the experiment. Hence, the results presented in Figure 8b feature a significant spread of velocity values as well as a diversified sense of velocity vectors. The maximum velocity of approx. 0.003 m/s is 10 times smaller than the value recorded for the outermost reactor areas during mixing. For such a value the maximum Re number is 300, which indicates that the system is considered laminar. While in the case of variant 17 the maximum Reynolds number exceeded 50,000, which classifies the system as a turbulent one. In the both reactors we observe significant gradient in the fluid velocity. Each reduction of velocity by 0.1 m/s means Reynolds number decreasing by 10,000 and 14,000, respectively for reactor no. 1 and reactor no. 2. The Reynolds number was defined by Equation (1):

(1) Re=uLν

where: *u*—local velocity of the solution, *ν*—kinematic viscosity of the solution. The characteristic dimension *L*, in the Reynolds number, was described by the reactor base diameter. The NaOH kinematic viscosity was 8.82 × 10^−7^ m^2^/s.

The fundamental chemical reaction of Sn dissolution in the NaOH solution is [21]:

2Sn + 2OH^−^+O_2_ = 2HSnO_2_^−^ (2)

The leaching agent was sampled during experiments to verify the observed hydrodynamic relationships and their impact on tin leaching. The obtained values of tin concentration in the leaching solution (in appropriate time intervals), expressed as arithmetic mean values of four simultaneous measuring series, are presented in Figure 9. Results in Figure 9a confirm that the leaching was most efficient in the case of obtaining the highest velocity in the system. However, it should be remembered that in case no. 17, the process was carried out at a temperature of 294 K, at which an additional chemical reaction decided about the pace of tin dissolution. Instead, the results related to powder show clearly that an appropriate grain size ensured to the leached material can intensify the leaching even 20 times. The grinding of material and thereby enlarging the contact boundary is efficient even in the area of natural convection, where the supply of tin in a powder form resulted in a fivefold increase in the process efficiency as against the reactor with an active mechanical mixer and a rotational speed of 500 rpm.

## 4. Conclusions

Based on the carried out laboratory experiments, it was found that:

▪ The PIV method can be successfully applied to search for new solutions in the field of hydrometallurgy and to optimize the leaching processes, among others;
▪ A parabolic velocity distribution developing in the considered reactors mixed by means of the mechanical propeller was detected. For the highest rotational speed of the propeller, nearly 50% reduction of fluid velocity was obtained in the zone of 70% distance from a propeller axis;
▪ The hydrodynamic structures developing in the working space of a considered batch of reactors are affected by the type of continuous phase and the leached phase form. The difference in the maximal velocity between the distilled water system and NaOH-Sn powder system amounted to nearly 0.34 m/s for the same propeller speed, propeller blades position and reactor type;
▪ The leaching is affected by the reactor geometry, leached phase form and the hydrodynamic structure developed in its working volume, especially when a convectional or diffusion mass transport decides the process efficiency. Hence appropriate preparation of the leached material, intensifying its dispersion in the leaching agent volume as well as mixer’s direct impact zones, is essential.

## Figures and Tables

**Table 1:** Research cases for water and both reactors.

Case No.	Temperature, K		Propeller Speed, Rpm			Propeller Blades Position	
	294	323	50	300	500	Bottom	Top
1	×	−	×	−	−	×	−
2	×	−	−	×	−	×	−
3	×	−	−	−	×	×	−
4	×	−	×	−	−	−	×
5	×	−	−	×	−	−	×
6	×	−	−	−	×	−	×
7	−	×	×	−	−	×	−
8	−	×	−	×	−	×	−
9	−	×	−	−	×	×	−
10	−	×	×	−	−	−	×
11	−	×	−	×	−	−	×
12	−	×	−	−	×	−	×

**Table 2:** Research cases for Sn–NaOH system and propeller speed 500 rpm.

Case No.	Reactor No.		Sn Form		Temperature, K		Propeller Blades Position	
	1	2	Powder	Drop	294	323	Bottom	Top
13	×	−	−	−	×	−	×	−
14	×	−	−	−	×	−	−	×
15	−	×	−	−	×	−	×	−
16	−	×	−	−	×	−	−	×
17	×	−	−	×	×	−	×	−
18	×	−	−	×	−	×	×	−
19	−	×	−	×	×	−	×	−
20	−	×	−	×	−	×	×	−
21	×	−	×	−	−	×	−	−
22	×	−	×	−	−	×	×	−

## Data Availability

All data are included in the paper.
